# Simulation Study on the Defect Generation, Accumulation Mechanism and Mechanical Response of GaAs Nanowires under Heavy-Ion Irradiation

**DOI:** 10.3390/nano12040611

**Published:** 2022-02-11

**Authors:** Tongxuan Jia, Zujun Wang, Minghua Tang, Yuanyuan Xue, Gang Huang, Xu Nie, Shankun Lai, Wuying Ma, Baoping He, Shilong Gou

**Affiliations:** 1 School of Materials Science and Engineering, Xiangtan University, Xiangtan 411105, China; 201921001450@smail.xtu.edu.cn (T.J.); 202021001704@smail.xtu.edu.cn (G.H.); 202021001734@smail.xtu.edu.cn (X.N.); 202021001713@smail.xtu.edu.cn (S.L.); 2 State Key Laboratory of Intense Pulsed Irradiation Simulation and Effect, Northwest Institute of Nuclear Technology, Xi’an 710024, China; xue_yuanyuan@nint.ac.cn (Y.X.); mawuying@nint.ac.cn (W.M.); hebaoping@nint.ac.cn (B.H.); goushilong@nint.ac.cn (S.G.)

**Keywords:** GaAs NWs, defect mechanisms, molecular dynamics, mechanical properties, defects

## Abstract

Nanowire structures with high-density interfaces are considered to have higher radiation damage resistance properties compared to conventional bulk structures. In the present work, molecular dynamics (MD) is conducted to investigate the irradiation effects and mechanical response changes of GaAs nanowires (NWs) under heavy-ion irradiation. For this simulation, single-ion damage and high-dose ion injection are used to reveal defect generation and accumulation mechanisms. The presence of surface effects gives an advantage to defects in rapid accumulation but is also the main cause of dynamic annihilation of the surface. Overall, the defects exhibit a particular mechanism of rapid accumulation to saturation. Moreover, for the structural transformation of irradiated GaAs NWs, amorphization is the main mode. The main damage mechanism of NWs is sputtering, which also leads to erosion refinement at high doses. The high flux ions lead to a softening of the mechanical properties, which can be reflected by a reduction in yield strength and Young’s modulus.

## 1. Introduction

Compared with traditional bulk materials, semiconductor nanowires (NWs) on the one-dimensional nanoscale have excellent mechanical and optoelectronic properties [1,2] due to their remarkable quantum size effect and surface effect. With the development of metal-organic vapor phase epitaxy (MOVPE) technology, many examples of high purity and quality semiconductor NWs produced in practical experiments [3,4,5,6], which makes it possible to provide potential applications in the field of space-based nano-optoelectronic devices. Among these materials, III-V compounds such as gallium arsenide (GaAs) have excellent properties such as wide direct bandgap coverage [7] and high electron mobility [8]. Thus, GaAs NWs have gradually replaced silicon (Si), widely used in terahertz photodetectors, photovoltaic cells [1,9] and other fields. It is worth noting that GaAs NWs will inevitably suffer from heavy-ion irradiation in the synthesis, fabrication, characterization and normal devices’ operation [4,5], thereby changing the basic properties. For instance, the doping of the surface microstructure and the alternating of the chemical position can be realized under the ion-implanted impurity. On the other hand, similar to neutrons, heavy-ion displacement cascade collisions within the target materials can cause point defects, defect clusters and amorphous pockets, and eventually form steady-state damage configurations during long migration and recombination [10,11,12]. These steady-state defects on the surface and interior of NWs can degrade the mechanical properties of the devices, which may form hidden troubles such as mechanical failure and fracture too early during the space engineering mission. Therefore, the irradiation effect and mechanical response change of nanowires have become the active field in the modern nanowire field. Meanwhile, this has important implications for assessing radiation damage and the continued progress of radiation tolerant reinforcement.

Several research conclusions have been made in recent years regarding the irradiation damage of GaAs NWs. For instance, the enhanced sputtering damage mechanism is a distinct damage feature in high-density interfacial structures such as NWs compared to bulk structures, a view confirmed by Johannes et al. They also found through experimental and simulation methods that GaAs NWs under high fluencies of Mn ion beam show erosion refinement phenomena and nonlinear increase of doping [13]. Afterward, Li et al. carried out 1 MeV proton and H^+^ irradiation experiments on GaAs/AlGaAs core-shell NWs at room temperature and found that the minority carrier lifetime is closely related to the irradiation-induced defect density by photoluminescence (PL) method. In addition, the size dependence of the carrier lifetime damage factor of GaAs NWs is mainly attributed to a special dynamic mechanism of defect annihilation, as summarized by them [14,15]. These results generally indicate that NW is a better candidate structure for radiation resistance. In addition to these cases, structural transitions of amorphization or transition to another crystalline state usually occur in irradiated nanostructured materials. Amorphization and cubic-hexagonal transition phenomena [16,17] of Si NWs under irradiation conditions were found by Dai et al. and Rodichkina et al., while Alekseev et al. [18] also showed that radiation leads to amorphization in ZB/WZ heterostructure GaAs NWs, where the WZ structure transforms into β-Ga_2_O_3_ crystals. Meanwhile, there is continuous progress in the study of the mechanical properties of irradiated GaAs NWs. Borschel et al. [19] found a certain bending degree of GaAs NWs with high-energy ion conditions. Finally, they pointed out that defects such as vacancies or interstitials created by displacement cascades are the key cause of deformation. In general, the defect generation and structural changes of irradiated NWs have a significant impact on their properties, and therefore exploring the damage mechanism is an important guide for the design and preparation of nanowire materials. However, so far, there are few or almost no studies on the defect generation, accumulation mechanism and mechanical response of GaAs NWs under heavy-ion irradiation.

Although some advanced modern experimental instruments such as the transmission electron microscopy (TEM) [20,21] can identify specific defects of materials, there are some shortcomings and limitations in observing the complete process and complex mechanism of multi-cascade defects interaction at the micro-scale. So far, most of the studies on the origin of radiation tolerance of nanomaterials have focused on computational studies. Molecular dynamics (MD) simulations can enable complex mechanisms at the microscopic atomic scale that are difficult to reveal by practical experiments and provide unique insights into experiments [22,23,24]. Now, MD simulation is widely used to simulate irradiation and mechanical effects of semiconductor (GaN [25] and Si [11]) and metal (Au) [20,26,27] NWs. Here, we propose the MD simulation method to study the defect generation, accumulation mechanism and mechanical response of irradiated GaAs NWs under the heavy-ion beam. The paper is organized as follows: In Section 2, we present a method for simulating ion radiation and mechanical stretching. In Section 3, we simulate the defect generation and damage accumulation mechanism of GaAs NWs in terms of single ion and ion beam damage. Then, the nano-effects of damage accumulation and specific point defects on GaAs NWs mechanical properties are pointed out through tensile fracture simulation.

## 2. Methodology

**Figure 1:**
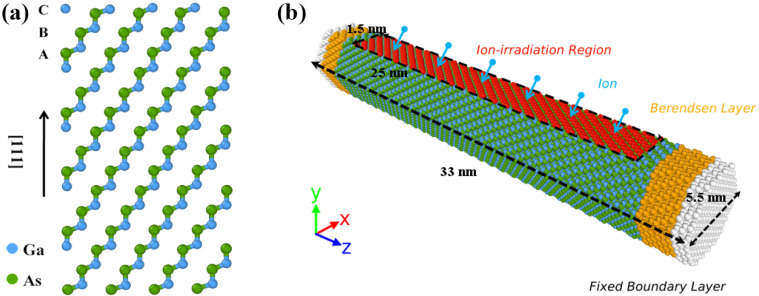
The schematic diagram of ZB-GaAs NWs. (**a**) Atomic configurations of three basic modules as A, B and C. (**b**) The computational model of irradiated GaAs NW, where the red, yellow and white regions represent irradiation, thermostat and fixed region, respectively.

In this paper, we use the MD software called Large-Scale Atom/Molecule Massive Parallel Simulator (LAMMPS) [28] to simulate the whole process of ion irradiation and tensile deformation. For MD simulations, as shown in Figure 1a, the lattice constant of the ZB GaAs model is 5.653, where the ordered sequence is tightly combined by three basic tetrahedral bonding modules including C, B and A. Each module contains a Ga-As pair. Furthermore, a complete synthesis model of GaAs NW including 29376 atoms with the [111] axis and six side facets [29] is shown in Figure 1b, where the X, Y and Z axes are oriented along [11–2], [1–10] and [111] directions, respectively. The longitudinal length of NWs is about 33 nm and the cross-sectional diameter is about 5.5 nm to maintain the aspect ratio of 6:1 [30,31].

### 2.1. Simulation of Ion Implantation

After creating the GaAs NW mentioned previously, the objective structure needs to relax for up to 100 ps to reach a steady state, where the temperature and pressure meet the normal experimental requirements at 300 K and 0 Gpa pressure (close to vacuum), respectively. About the selection of heavy ions, we choose Ga ions, which are widely present in focused low earth orbit, as the energy particles in this case, because their radiation phenomena are more likely to occur in actual nanowire work [32]. Then, as shown in the irradiated GaAs NW model in Figure 1b, Ga ions at approximately 3 Å directly above the nanowire are randomly selected and given some energy for incidence in a planar region with an area of 1.5 × 25 nm^2^. It is worth noting that the ions are neutralized by charge transfer as they approach the surface, so the neutral atomic approximation of Ga ions is required. The periodic boundary condition is used along the axial direction, while X and Y directions are defined as free surfaces. The micro-canonical ensemble (NVE) is applied in the interaction between ions and nanowires. During this process, we continue our previous works by using the multiple-phase timestep procedure to simulate the whole high-energy particle collisions with a total time of 20.4 ps. Moreover, as shown in Figure 1b, a fixed region of three modules in length is set at both ends of GaAs NW to prevent movement due to ionic momentum. Meanwhile, Berendsen temperature control is applied to the area near the fixed region to release the cascade heat in time. Finally, we need to perform an annealing process in an isothermal isobaric (NPT) ensemble with a time of 30 ps to bring the target temperature down to 300 K. The relaxed structure is for the next ion implantation. The total ion number is 200 and the ion dose reaches 5.33 × 10^14^ ions/cm^2^.

### 2.2. Simulation of Tensile Deformation

In terms of mechanical response, we carry out uniaxial tensile simulations of irradiated GaAs NWs, and GaAs NWs containing each type of point defects, respectively. Before the simulation starts, the energy of the nanowire system needs to be minimized through the conjugate gradient method, followed by a 30 ps dynamic equilibration at 300 K under NPT ensemble. At this point, the axial pressure on NWs has been removed. Subsequently, the tensile strain is loaded along the z direction of this system, while the strain rate is kept at 1 × 10^−3^ ps^−1^. In addition, NPT ensemble continues and the periodic boundary condition is applied in all directions. During the process of each strain increment, the calculation of stress is made by averaging over 2000 steps. The stress level between each atom is calculated by the virial stress theorem [33], which is given by the following equation:

(1) $$\sigma_{z}^{i}(\varepsilon_{z})=\frac{1}{V_{i}}\left[m_{i}v_{z}^{i}v_{z}^{i}+\frac{1}{2}\sum_{j(\neq i)}^{N}F_{z}^{ij}(\varepsilon_{z})r_{z}^{ij}(\varepsilon_{z})\right]$$

where *ε_z_* and *σ_z_* represent the normal strain and virial stress along the axial direction, $v_{z}^{i}$ and *m*_i_ refer the velocity and mass of atom *i*, $F_{z}^{ij}$ and $r_{z}^{ij}$ are the interatomic force and displacement between atoms along the z direction. According to this formula, we derive the stress-strain curves for GaAs NWs in the above required environment. In addition, the maximum stress in the stress-strain curve represents the yield strength.

### 2.3. Interatomic Potentials

There are many versions of interatomic potential that can be described regarding the atomic interaction of GaAs, among which the precision of each interatomic potential with respect to specific properties is different. For instance, the Tersoff potential [34] can describe the physical properties of GaAs crystals more accurately, especially the aspect of the formation energy of point defects. However, at high energy conditions, the classical potential cannot be used to its advantage because of the lack of sufficiently short interactions. To solve this problem, the ZBL potential [35], which can be used to modify the interatomic repulsive ability by a screening function, is used to form a hybrid potential by connecting with the long-range potential at a distance of less than 1 Å. At present, the Tersoff/ZBL hybrid potential has become a common potential function used to simulate displacement cascade collisions. For the simulation part of ion implantation, we have adopted the hybrid potential by Albe et al. [36] and Gao et al. [10] In terms of the tensile deformation simulations, we use the Vashishta potential function which is very close to the experimental value in describing the elastic constants of the GaAs crystal [37]. This potential function has been used several times to simulate the stretching and compression of GaAs NWs, showing a series of mechanical characteristics such as size effect, brittle to ductile transition, and self-healing phenomena, all of which have been demonstrated in the experiment [30,31]. The parameters related to the Vashishta potential are taken from Ref. [37].

### 2.4. Analysis Method

In the present work, the visualization software Ovito [38] is used to observe and analyze the whole simulation process. Among them, we extract and analyze point defects such as vacancies and interstitials using the Wigner-Seitz defect analysis method, the principles of which are similar to our previous work [22,23]. Additionally, sputtered atoms are defined as arbitrary atoms located 3 Å away from the surface. Subsequently, for the aspect of defect structure, the Identify diamond structure (IDS) analysis module in Ovito is used to extract the GaAs defect structure at each stage. Dislocation extraction analysis (DXA) is also used to analyze the location and nature of dislocations in GaAs NWs.

## 3. Results and Discussion

### 3.1. Single Ion Damage Effects

**Figure 2:**
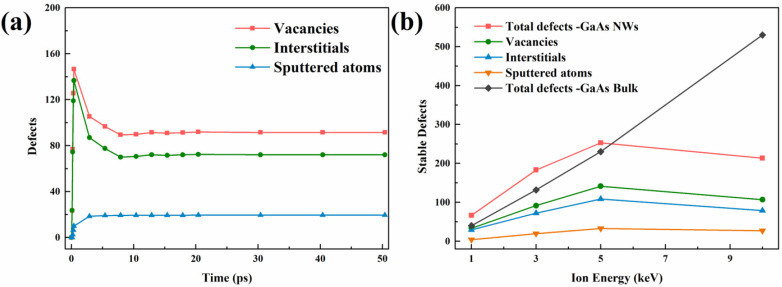
The defect number of irradiated ZB-GaAs NWs under single ion damage condition. (**a**) Defect evolution for each type as a function of time under 3-keV single ion damage. (**b**) The number of stable defects for each type as a function of ion energy.

For the study of defect generation mechanisms, the analysis was performed from the perspective of single ion damage. The simulation results are obtained by averaging the results of thirty different and independent single ion implantations, in which the selection of ions is random and the energy range from 1 to 10 keV. The evolution of defects in irradiated bulk structures is generally characterized by a trend of “rise, down and steady”, which is demonstrated in our previous work [22,23]. However, NWs exhibit new features. The results of defect evolution at an ion energy of 3 keV are shown in Figure 2a. We can see that the number of vacancies and interstitials is different although they appear similar trend as the bulk. This is attributed to the fact that NWs have a unique mechanism of sputtering damage, where many surface atoms lose their interaction with other atoms due to the bombardment of ions and start to detach from the surface. In addition, the number of sputtered atoms gradually stabilizes with the appearance of the thermal peak, thereby showing an overall trend of “rise and steady”.

**Figure 3:**
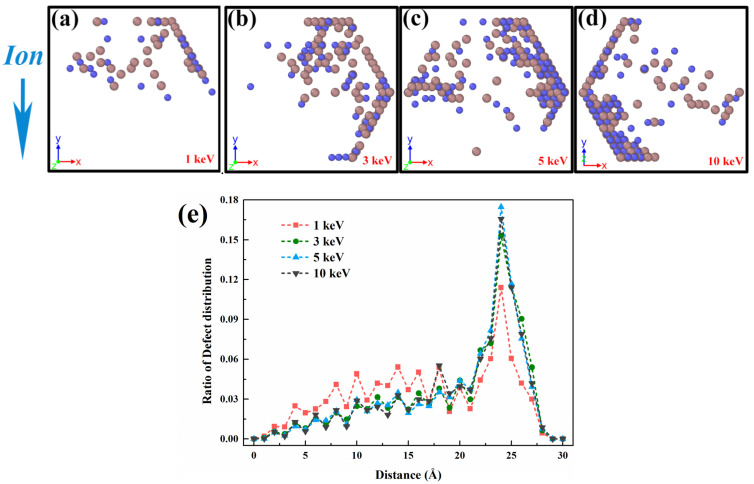
The defect distributions of irradiated ZB-GaAs NWs under single ion damage conditions. (**a**–**d**) Defect distribution diagram with ionic energy range in 1 to 5 keV. (**e**) Ratio of defect distribution as a function of distance.

In addition, in Figure 2b, we also investigate the results of the number of steady-state defects as a function of ionic energy and find that at low energies, the total number of defects in NWs is larger than in the bulk. Much of the reason for such a result is attributed to the surface effect of NWs. Interstitial atoms from ion collisions have a higher migration rate compared to vacant atoms and automatically migrate to the surface to form adsorbed atoms, which eventually leads to a situation where the total number of defects is larger than the bulk materials. To further illustrate this phenomenon, we analyzed the defect distribution under the same cross-section. The surface effect can also be seen directly in Figure 3. As shown from Figure 3a–d, as the ion energy increases, more and more defects keep migrating outward and aggregating. Figure 3e illustrates more directly the greater proportion of defects away from the center at high energies. At this point, the defect distribution is characterized as empty in the center and dense around. However, instead of showing a linear increase in the number of steady-state defects in NWs with increasing ion energy as in the bulk structure, there is a trend of a nonlinear increase followed by a decrease. This is because, in shorter diameter NWs, the overall stopping power of the structure against energetic particles is lower and fewer collisional energy transfers occur, leading to this phenomenon. This result was also found in GaN [39] and the same configuration SiC [32]. A moderate increase in the incidence angle and nanowire diameter can increase the probability of collisions, which is the focus of future work.

### 3.2. High-Doses Ion Irradiation Effects

**Figure 4:**
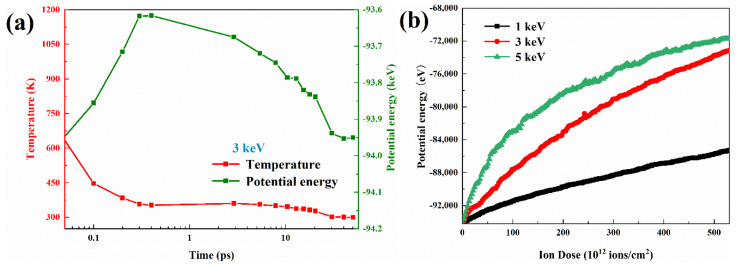
Changes in system properties during ion damage. (**a**) Evolution of the system temperature and energy with time under 3 keV single ion effect. (**b**) Energy evolution of the system under high-dose ion implantation.

The total energy and temperature post-temporal evolution of the system under 3 keV single Ga ion damage is shown in Figure 4a. It can be seen that the temperature is at peak at the instant of single ion damage, then immediately decreases under the effect of heat dissipation from the thermostat and atomic compounding, and finally converges to the target stable value of 300 K. At the same time, the system energy profile gradually rises to its peak and then also decreases. It is noteworthy that there is a large increase of system energy from the initial state.

**Figure 5:**
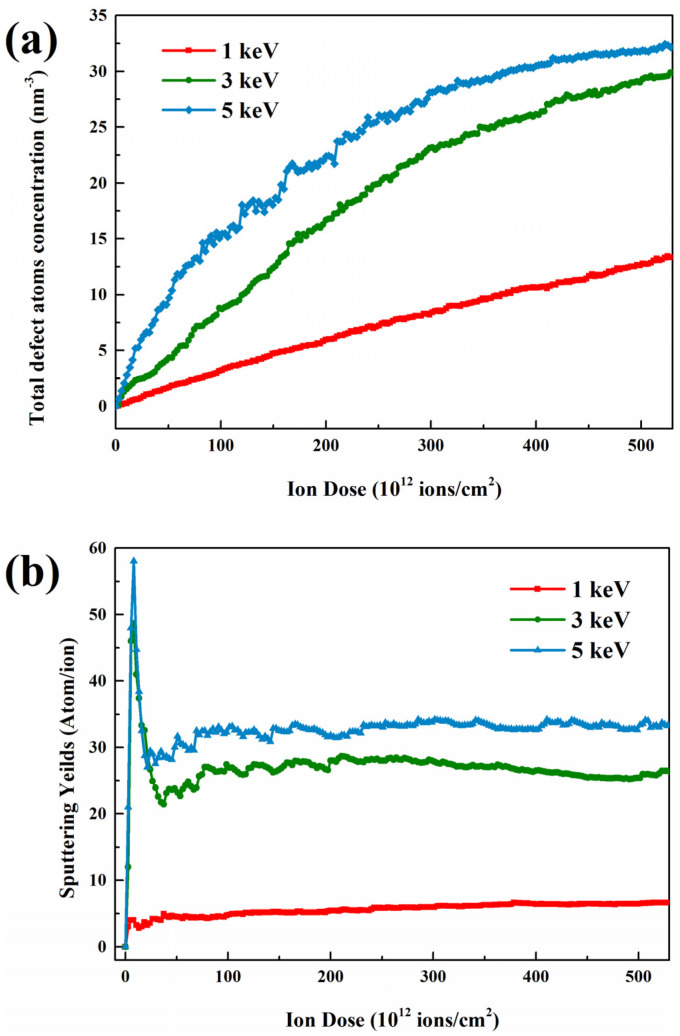
Evolution of (**a**) the total defect atoms concentration, (**b**) the sputtering yields as a function of ion dose.

To analyze the defect accumulation mechanism of irradiated GaAs NWs, the evolution of defect density as a function of ion doses is expressed in Figure 5a. The results show that the defects accumulation rapidly at low ion doses and start to show saturation with continuous ion injection, which is more pronounced at high energies. The evolution of the defect density shows a similar trend to the system energy profile in Figure 4b. We attribute this change to the natural competition and annihilation; where more defects migrate to the nanowire surface, undergo compounding and disappearance behavior. And thus, it can maintain a dynamic equilibrium, which is different from the way of accumulation in bulk materials. Although the damage mode of other nanowires is different, they also show a similar manifestation. As the main damage mode for smaller NWs, the variation of sputtering yield is shown in Figure 5b. It can be seen that the sputtering yield increases with increasing ion dose and later decreases to a stable value with the energy separation of the amorphous layer [40].

**Figure 6:**
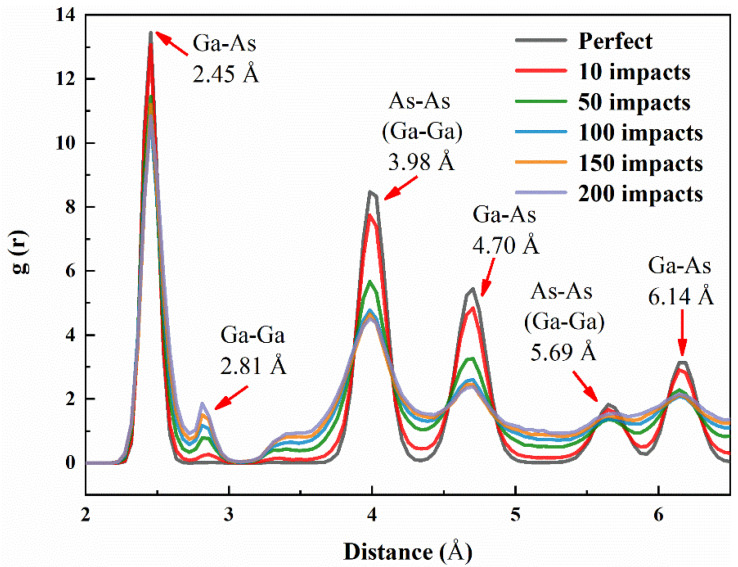
Radial pair distribution functions (RDFs) of GaAs NWs in a major damage area a function of 3 keV ion impacts.

In the case of ion high-dose irradiation of GaAs NWs, we also investigate the effect of the ion beam on the structural transition. Studies on the orderliness of the damage region at sustained ion doses make use of the radial distribution function (RDF) [41], which expresses the probability of finding another atom within a certain range of a given atom. The formula is expressed as follows:

(2) $$dn(r)=\frac{N}{V}g(r)4\pi r^{2}dr$$

where *N* is the total number of atoms in the system and *V* represents the volume of the selected area. The results of the RDF analysis of the structures at different irradiation doses are shown in Figure 6. The appearance of Ga-Ga bonds may suggest the formation of Ga defect clusters. In general, the gradual flattening of the pair correlation function curve implies the appearance of amorphous structures. This is in agreement with numerous experimental results. In particular, the lengths of the Ga-As and As-As (Ga-Ga) bonds before irradiation were evaluated by calculation of the distribution function, and they were 2.45 and 3.98 Å. This is the same value as that obtained by Chung et al. [42]

**Figure 7:**
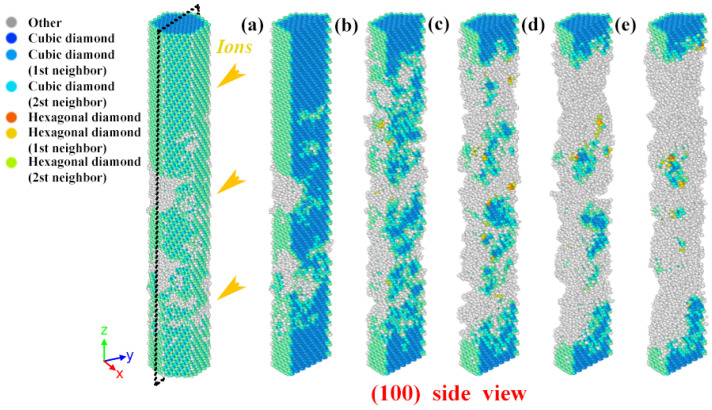
Schematic diagram of the cross-sectional structural transition with 3 keV ion impactions. (**a**–**e**) are at 0, 133, 266, 400, 533 × 10^12^ ions/cm^2^, respectively.

**Figure 8:**
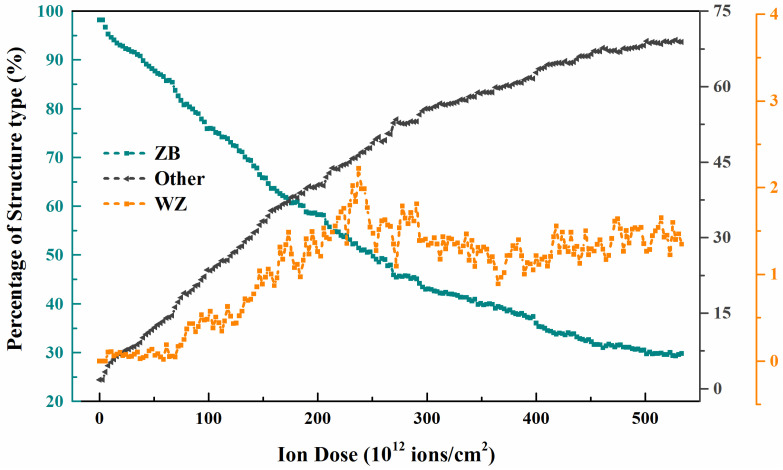
Evolution of percentage of structure type as a function of ion dose under 3 keV ion condition, blue, black and orange curves represent ZB, disorder and WZ structures, respectively.

As shown in Figure 7, (100) cross-sectional view is selected to observe the structural transformation process at high-dose ions. Under 3 keV ion damage, the nanowires will transform gradually from the normal cubic diamond (ZB) structure to disorderly, i.e., amorphous. The degree of amorphization becomes progressively more severe with the increase in the number of injected ions. This condition is also shown in Figure 8, where it can be seen that ZB-amorphization is the predominant mode. And the amorphization transition rate is also from fast to slow, with the ratio from 0 to 68.9%, and finally the amorphous region at high dose shows a stable state. The difference is that the percentage of ZB structure shows a trend opposite to that of amorphization. This is consistent with the mechanism of defect accumulation as discussed previously. At this time, as pointed out in Figure 7, there are many sputtered atoms detached from the nanowire surface under constant ion injection. This leads to a continuous erosion refinement in the damage region, which is consistent with previous experimental results [13]. Notably, in Figure 7, our simulations also demonstrate another form of structural transition in irradiated GaAs NWs. Combining Figure 7 and Figure 8; we find a small amount of atoms to hexagonal diamond (WZ) structural change region in GaAs NWs at high dose damage, and the same structural transformation phenomenon was also exhibited in irradiated Si [17] and 3C-SiC [43]. This conversion pattern shows an upward to stable trend.

### 3.3. Tensile Deformation of Irradiated GaAs NWs

**Figure 9:**
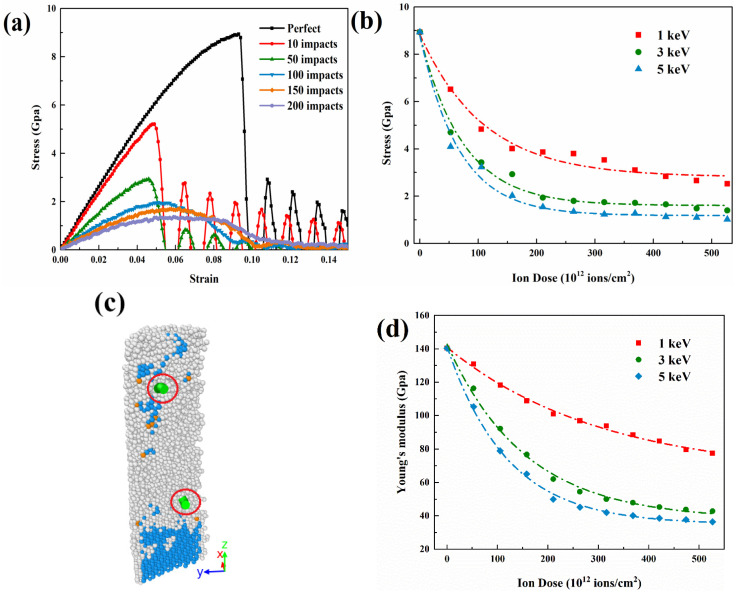
Simulation of tensile deformation of irradiated GaAs NWs. (**a**) Tensile stress-strain curves under different 3 keV ion impacts. (**b**) Maximum tensile strength, (**c**) Schematic diagram of dislocation structure and (**d**) Young’s modulus as a function of ion dose.

In the environment of ion irradiation, GaAs mechanical properties undergo will continuously change. In this section, we use uni-axial tensile in the [111] direction to calculate the stress-strain curves of un-irradiated and irradiated GaAs NWs to evaluate the mechanical properties under the irradiation effect. Here, five state points with the number of injected ions of 0, 50, 100, 150 and 200 are taken. The stress-strain curves are shown in Figure 9a. First, under the unirradiated condition, the continuous stretching causes increased stress, and NWs go through the elastic and yield stages, respectively. After reaching the maximum value, i.e., yield strength, the stress value suddenly decreases, and the fracture stage is then. The tensile strength and strain values obtained therein are very close to the simulated data from Wang et al. [30]. Subsequently, the yield strength of the nanowires shows a weakening trend with the deepening of the irradiation level. As depicted in Figure 9b, it can be seen that the higher energy of the ion beam has a greater effect on the intensity. The effect of ion dose decreases nonlinearly throughout the irradiation process and eventually stabilizes. Furthermore, it is noteworthy that the nanowires exhibit an extended deformation behavior, which is due to two reasons. First, the presence of point defects such as vacancies and interstitial atoms created in the damaged region can weaken the atomic interactions and eventually lead to material softening [44]. This is a factor that has been confirmed by many studies. Secondly, as shown in the partial cross-sectional structure in Figure 9c, nanostructures that are under high ion-dose appear as 1/6<112> dislocation types, whose motion ensures plastic deformation. It is also demonstrated in the study of Wang et al. [31] In terms of Young’s modulus, Figure 9d shows that the mechanical parameters usually decrease with increasing dose, reaching saturation at doses greater than 4 × 10^14^ ions/cm^2^ at 5 keV energy. Point defects and small clusters induce changes in the mechanical properties of GaAs NWs at low ion doses, while a crystalline to amorphous (c-a) transition occurs at high doses, saturating the mechanical properties. Overall, the results point out that the radiation effect has a great influence on the mechanical properties of GaAs NWs.

**Figure 10:**
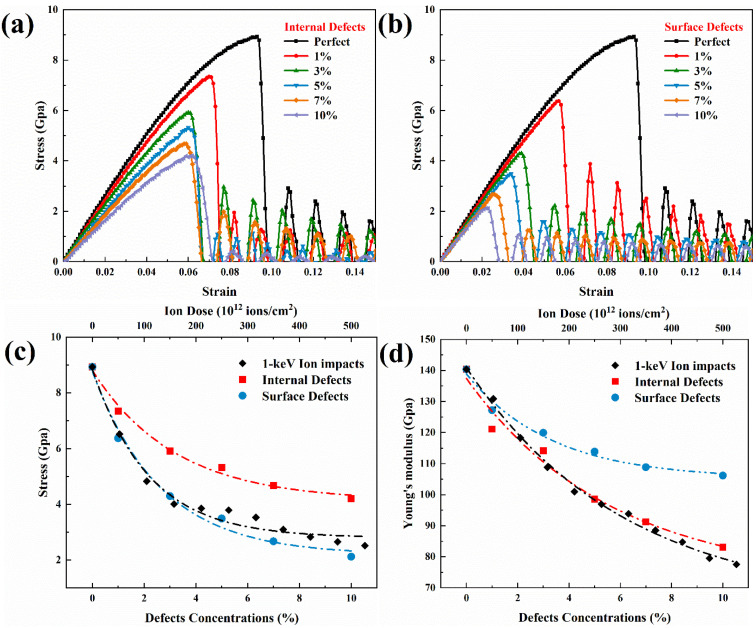
Simulation of tensile deformation of irradiated GaAs NWs under different type defects. Tensile stress-strain curves of (**a**) internal defects and (**b**) surface defects under different defect concentrations. (**c**) Tensile strength and (**d**) Young’s modulus as a function of defect concentrations.

As discussed previously, defects are the main cause of the variation in the mechanical properties of irradiated GaAs nanowires. However, in this setting, the effects of different types of defects, such as internal and surface defects, are different due to the special structural nature of the high-density interfaces of nanowires [45]. In this paper, vacancy defect configurations with concentrations of 1%, 3%, 5%, 7% and 10% are constructed inside and on the surface of GaAs nanowires by randomly deleting atoms, respectively. Furthermore, because the number of defects at 1 keV ion dose is similar to the defect concentration, this ion irradiation model is used for comparison. After that, unidirectional stretching simulations were performed along the Z-axis with the same environmental setting conditions as before. The simulation results are shown in Figure 10. It can be seen that with increasing defect concentration, both internal and surface defects affect the yield strength and critical strain. In terms of tensile strength, surface defects have a more pronounced effect than internal defects. However, for Young’s modulus, the situation is reversed. The internal defects play an important role in weakening the ability of nanowires to resist deformation. A large part of this is due to the fact that vacancy defects formed in the interior cause more atomic bonds to break. As the concentration of defects increases, there is a greater probability that large clusters of vacancies will form, resulting in a reduction in Young’s modulus. However, the mechanical properties tend to the saturation stage with increasing concentration for both surface and internal defects.

## 4. Conclusions

In this paper, we use MD simulations to reveal the defect generation, accumulation mechanism and mechanical response changes of GaAs NWs under heavy-ion irradiation. In terms of defect generation, sputtering damage of NWs is the main damage. What is more, the presence of surface effects at lower energies makes the number of nanowire defects larger than that of bulk structures. The low stopping power of the nanowire structure for high-energy particles means that less collisional energy transfer occurs, which is the main reason for the non-linear relationship of the defects. In terms of defect accumulation, defects exhibit a special mechanism of rapid accumulation to saturation, which is closely related to the surface dynamic annihilation process. Under the continuous ion injection, GaAs nanowire structure transformation mode is mainly an amorphous pathway, when the nanowire damage region shows erosion refinement phenomenon. In addition, there is also a small amount of ZB atoms transformed into WZ structures. The high flux ions lead to a softening of the mechanical properties, which can be reflected by a reduction in yield strength and Young’s modulus. Among them, for yield strength, internal defects have a greater impact than surface defects, but for Young’s modulus, this situation is reversed. 

## Data Availability

The raw/processed data required to reproduce these findings cannot be shared at this time as the data also forms part of an ongoing study.
